# A dual nodal response to the parahisian pacing and induction of the retrograde right bundle branch block maneuvers

**DOI:** 10.1002/joa3.12474

**Published:** 2020-12-07

**Authors:** Ahmet Korkmaz, Tolga Cimen, Meryem Kara, Bulent Deveci, Ahmet Lutfu Sertdemir, Ozcan Ozeke, Serkan Cay, Firat Ozcan, Serkan Topaloglu, Dursun Aras

**Affiliations:** ^1^ Department of Cardiology Ankara City Hospital University of Health Sciences Ankara Turkey; ^2^ Department of Cardiology Dışkapı Yıldırım Beyazıt Training and Research Hospital University of Health Sciences Ankara Turkey; ^3^ Department of Cardiology Necmettin Erbakan University Meram Medical Faculty Konya Turkey

**Keywords:** atrioventricular nodal reentrant tachycardia, atrioventricular node, Parahisian pacing, PHP, retrograde right bundle branch block

## Abstract

We presented intracardiac electrograms during the parahisian pacing, which represent three types of retrograde conduction and focus on the mechanism of types of retrograde conduction on wide QRS complexes and conclude that the two types of QRS of the retrograde conduction resulted from the presence or absence of retrograde block at the right bundle branch.

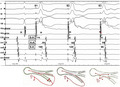

## 1 EPS FOR RESIDENT PHYSICIANS

**FIGURE 1:**
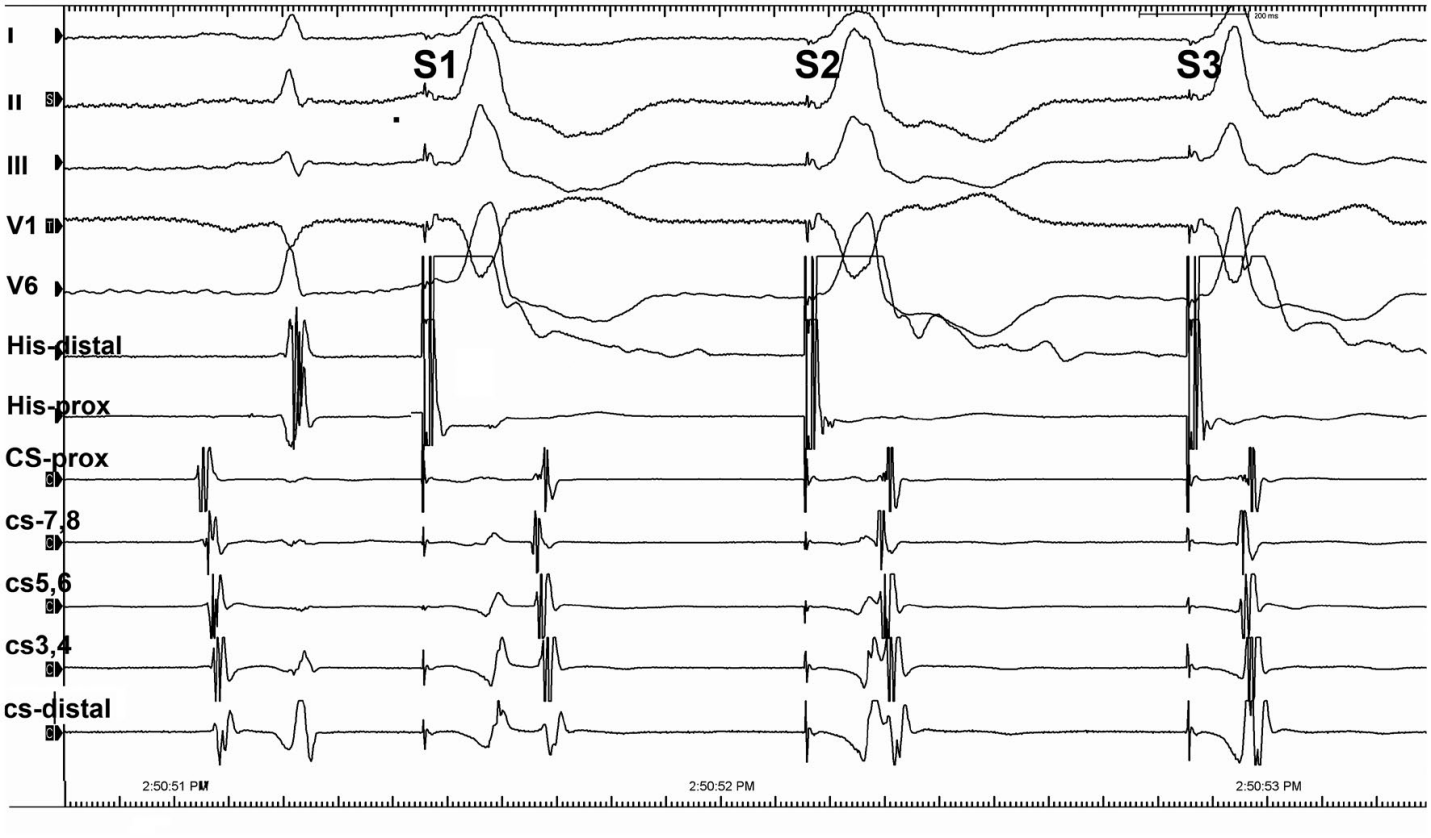
Surface and intracardiac electrograms during the parahisian pacing maneuver

A 53‐year‐old man presented with repeated paroxysms of palpitations resulting from a rapid narrow QRS complex tachycardia. The electrocardiography during sinus rhythm did not exhibit any delta waves. Parahisian pacing (PHP) was performed to discriminate between a retrograde septal accessory pathway (AP) and atrioventricular (AV) nodal conduction (Figure 1). What is the mechanism of the three different ventriculoatrial (V‐A) responses to a PHP maneuver?

**FIGURE 2:**
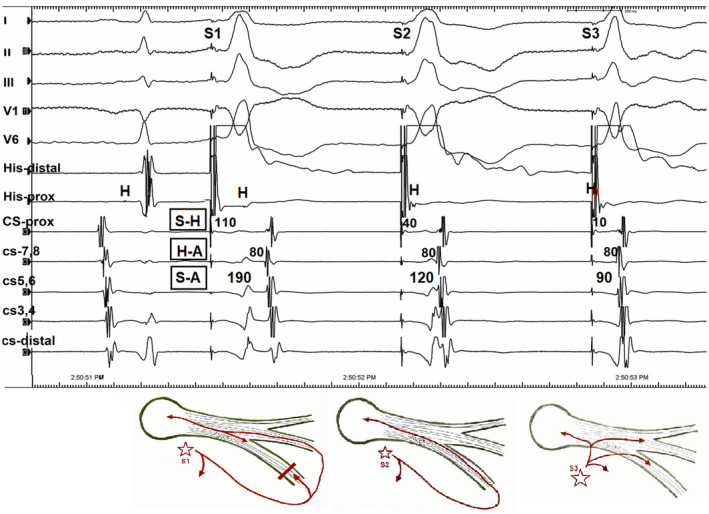
Surface and intracardiac electrograms during the parahisian pacing maneuver. It can be readily observed that the QRS complex on the right side (S3) is narrower than that on the left (S1) and middle (S2) side of the QRS complexes. Loss of direct His pacing is verified by the emergence of the retrograde His bundle deflection in the first (S1, left) and second (S2, middle) paced QRS complexes, giving further evidence that the His bundle has not been captured

The PHP is among the most useful maneuvers in cardiac electrophysiology when dealing with difficult cases. It is a handy strategy to distinguish between retrograde activation from the ventricle to the atrium over the septal AP or AV node.^1^, ^2^ Its complex behavior can create different patterns of activation depending on the pacing cycle length (CL)^1^, ^3^, ^4^; therefore, it should be performed at a constant CL (at CL 20‐30 ms shorter than the sinus CL) to avoid decrement/block within a His bundle (HB) system (HPS), AV node, or dual AV node pathways. Another maneuver, in some ways related to PHP, that can be useful to diagnose the nature of retrograde VA conduction is the induction of retrograde right bundle (RB) branch block (rRBBB).^5^ There are obvious similarities and important differences between the induction of rRBBB and the PHP. Whereas the detection of retrograde HB potential rather than relying on QRS changes is the key for correct interpretation in PHP maneuver,^5^ the induction of rRBBB is also manifested as the sudden appearance of a clearly discernible HB electrogram. Activation sequence is also as important as V‐A activation time when analyzing the effects of both maneuvers. Because of the change in the rate of pacing (coupling interval‐CI) with the rRBBB technique, a possibility of decremental conduction or block in AV node or an AP exists^5^; therefore, the rRBBB maneuver is useful only when there is continued VA conduction with a similar activation.^5^ In the current case, the constant right ventricular pacing at a CL of 700 msec showed intact VA conduction (Figure S1). During PHP, both the atrial activation sequence and the earliest atrial site at His catheter were the same in these three stimulated QRS beats. Loss of HB capture was confirmed by the widening of the QRS complexes on the surface electrocardiograms (Figure 1). When the stimulus to atrium (S‐A) interval increases caused by identical increases of the stimulus to His (S‐H) interval with the same atrial activation sequence, this confirms that the retrograde conduction was AV node dependent. The second QRS change [Stimulus 2 (S2) at Figure 1] was associated with a 30 ms increase of the S‐A interval caused by a 30 ms increase of the S‐H interval, whereas the first QRS change [Stimulus 1 (S1) at Figures 1 and 2] was associated with a 100 ms increase of the S‐A interval caused by a 100 ms increase of the S‐H interval, indicating retrograde conduction via the AV node (Figure 2). In the third stimulus [Stimulus 3 (S3) at Figures 1 and 2] causing a narrow QRS beat, the retrograde impulse propagated directly to HPS and finally to the AV node as the classical nodal response. Whereas this pattern represented conduction exclusively through the AV node (Figure S1), the further S‐A prolongation during the rRBBB by the S1 is also second proof of AV node dependence conduction, indicating that retrograde atrial activation is linked to HB/RB capture.^3^ Because of rRBBB, HB activation occurs only after transseptal conduction and retrograde activation of the left bundle; the retrograde His activation is delayed (the increase in S‐H interval) and the atrial activation is also delayed (the increase in the S‐A interval) by an equivalent amount of the retrograde S‐H interval and with no change in the activation sequence, confirming the retrograde activation via the AV node.^5^ In contrast, if an AP was responsible for retrograde conduction to the atrium, despite a significant delay in the S‐H time, the S‐A interval would be fixed, and H‐A interval could be negative.^5^ In current tracing, the H‐A intervals remained relatively unchanged in all these morphologies with the earliest retrograde atrial activation in the His areas, representing the retrograde fast pathway conduction (Figure 2). Therefore, the current tracing showed a double nodal response to two simultaneously occurring PHP and rRBBB maneuvers. There were subtle differences in the QRS morphology, and timing of ventricular deflections in the coronary sinus recordings between the first and the second QRS complexes, and these differences can be explained by the presence or absence of retrograde block at the RB. The unexpected one beat block in the retrograde RB may be considered to be compatible with a gap phenomenon, in which ventricular impulse fails to conduct via retrograde RB but conduction resumes even with the same CI of ventricular stimuli.^3^ Anatomic slow pathway ablation was performed for therapy of confirmed typical AV nodal reentrant tachycardia. Finally, we presented intracardiac electrograms during PHP, which represent three types of retrograde conduction and focus on the mechanism of types of retrograde conduction on wide QRS complexes, and concluded that the two types of QRS of the retrograde conduction resulted from the presence or absence of retrograde block at the RB branch.

## DISCLOSURES

None.

## Supporting information

Fig S1
